# Rapidly Progressive Coexistence of Gastric Antral Vascular Ectasia (GAVE) and Portal Hypertensive Gastropathy (PHG) Presenting With Iron Deficiency Anaemia: Successful Use of PuraStat in a Patient With Implantable Cardioverter-Defibrillator (ICD)

**DOI:** 10.7759/cureus.96729

**Published:** 2025-11-12

**Authors:** Mohamed Alafifi, Mariam Shata, Karim Heiba, Amany Emara

**Affiliations:** 1 Internal Medicine, Blackpool Victoria Hospital, Blackpool, GBR; 2 Acute Medicine, Royal Preston Hospital, Preston, GBR; 3 General Practice, Spring House Surgery, Bolton, GBR

**Keywords:** argon plasma coagulation (apc) contraindication, endoscopic haemostasis, gastric antral vascular ectasia (gave), gastrointestinal bleeding, implantable cardioverter-defibrillator (icd), iron deficiency anaemia (ida), portal hypertensive gastropathy (phg), purastat

## Abstract

Gastric antral vascular ectasia (GAVE), or "watermelon stomach", is an uncommon but important cause of chronic gastrointestinal blood loss and iron deficiency anaemia (IDA). Although associated with cirrhosis and portal hypertension, it is regarded as distinct from portal hypertensive gastropathy (PHG), and the two conditions rarely coexist. We report an 85-year-old woman with cirrhosis and multiple comorbidities who presented with severe symptomatic IDA (Hb 60 g/L). Remarkably, oesophago-gastro-duodenoscopy (OGD) performed just five months earlier had been entirely normal, yet repeat gastroscopy revealed rapidly developed GAVE and PHG with active bleeding. Endoscopic images demonstrated both GAVE and coexistent PHG, an uncommon finding. Haemostasis was achieved with PuraStat, chosen instead of argon plasma coagulation (APC) due to the presence of an implantable cardioverter-defibrillator (ICD). The patient required a blood transfusion and intravenous iron therapy, was stabilised clinically, and was discharged with structured follow-up. This case highlights the rapid evolution of GAVE, its potential to cause severe anaemia, the unusual coexistence of GAVE and PHG, and the successful use of PuraStat as a novel haemostatic option when APC is contraindicated.

## Introduction

Iron deficiency anaemia (IDA) is common in older adults and warrants a thorough gastrointestinal evaluation. While nutritional deficiency, colorectal malignancy, and diverticular disease are frequent causes, less common entities such as gastric antral vascular ectasia (GAVE) and portal hypertensive gastropathy (PHG) should also be considered, particularly in patients with chronic liver disease.

GAVE accounts for approximately 4% of non-variceal upper gastrointestinal bleeding [1,2]. It is characterised endoscopically by longitudinal erythematous stripes in the antrum, known as the "watermelon stomach" appearance [1]. Histology typically reveals dilated mucosal capillaries, fibrin thrombi, and fibromuscular hyperplasia [3]. GAVE is strongly associated with cirrhosis, portal hypertension, autoimmune disease, and chronic kidney disease but may occur independently [3].

PHG, in contrast, is a distinct but related mucosal abnormality found in portal hypertension. It typically affects the gastric body and fundus, displaying a mosaic or "snakeskin" pattern on endoscopy. PHG is often asymptomatic but can cause chronic blood loss and IDA. Although both GAVE and PHG may coexist in cirrhotic patients, they differ in pathophysiology, distribution, and management.

We present the case of an elderly woman with recurrent IDA, eventually diagnosed with GAVE on repeat gastroscopy after an initial normal study, in the context of underlying cirrhosis and features of PHG. This case highlights the diagnostic challenge and clinical overlap between these two entities.

## Case presentation

An 85-year-old woman was referred on the IDA fast-track pathway with a background of chronic kidney disease, atrial fibrillation (on rivaroxaban), ischaemic heart disease with percutaneous coronary intervention (PCI) to the left anterior descending (LAD) artery in 2019, hypertension, type 2 diabetes, bilateral hip replacements, spinal stenosis, osteoporosis, osteoarthritis, and a dual-chamber implantable cardioverter-defibrillator (ICD).

**Figure 1:**
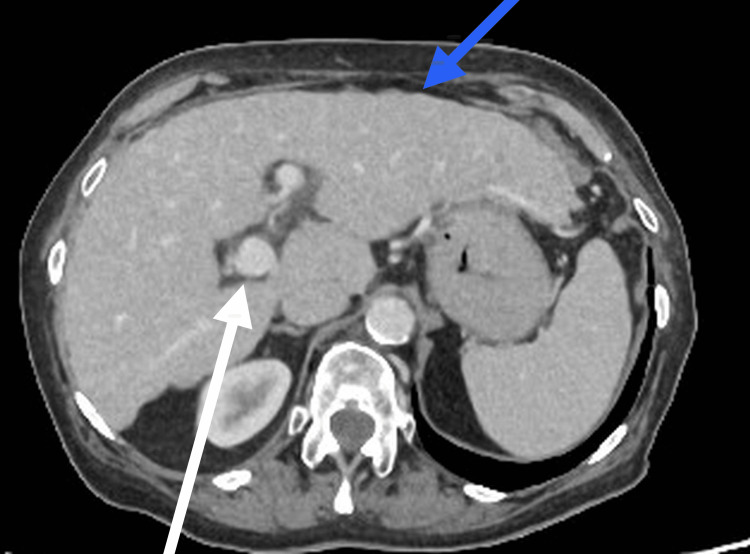
Contrast-enhanced CT of the abdomen showing nodular liver contour (blue arrow) with features of portal hypertension, including recanalisation of the umbilical vein (white arrow) CT: computed tomography

She had previously undergone a colonoscopy in 2020, which demonstrated diverticulosis and haemorrhoids, and in 2024, she had a repeat colonoscopy for a positive faecal immunochemical test (FIT) (>200), which excluded malignancy. Cross-sectional imaging (computed tomography (CT) of the thorax, abdomen, and pelvis in March 2025) had shown nodular liver margins, portal vein prominence, and recanalisation of the umbilical vein, consistent with cirrhosis and portal hypertension (Figure 1). Ultrasound of the abdomen in April 2025 demonstrated a coarse, irregular liver contour with preserved portal flow. A pelvic ultrasound showed no abnormalities aside from evidence of a previous hysterectomy. At the same time, a non-invasive liver screen was performed to exclude viral and autoimmune causes of liver disease; all results were negative. Based on this and her history of alcohol intake exceeding recommended limits, the cirrhosis was attributed to alcohol-related liver disease.

**Figure 2:**
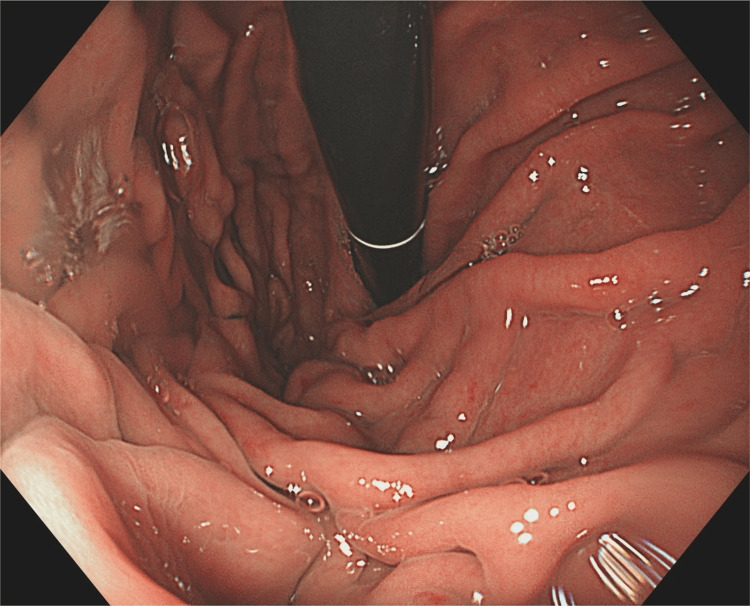
OGD (March 2025) showing a normal gastric mucosa without vascular lesions OGD: oesophago-gastro-duodenoscopy

In March 2025, she underwent an oesophago-gastro-duodenoscopy (OGD) for anaemia that was entirely normal (Figure 2).

**Figure 3:**
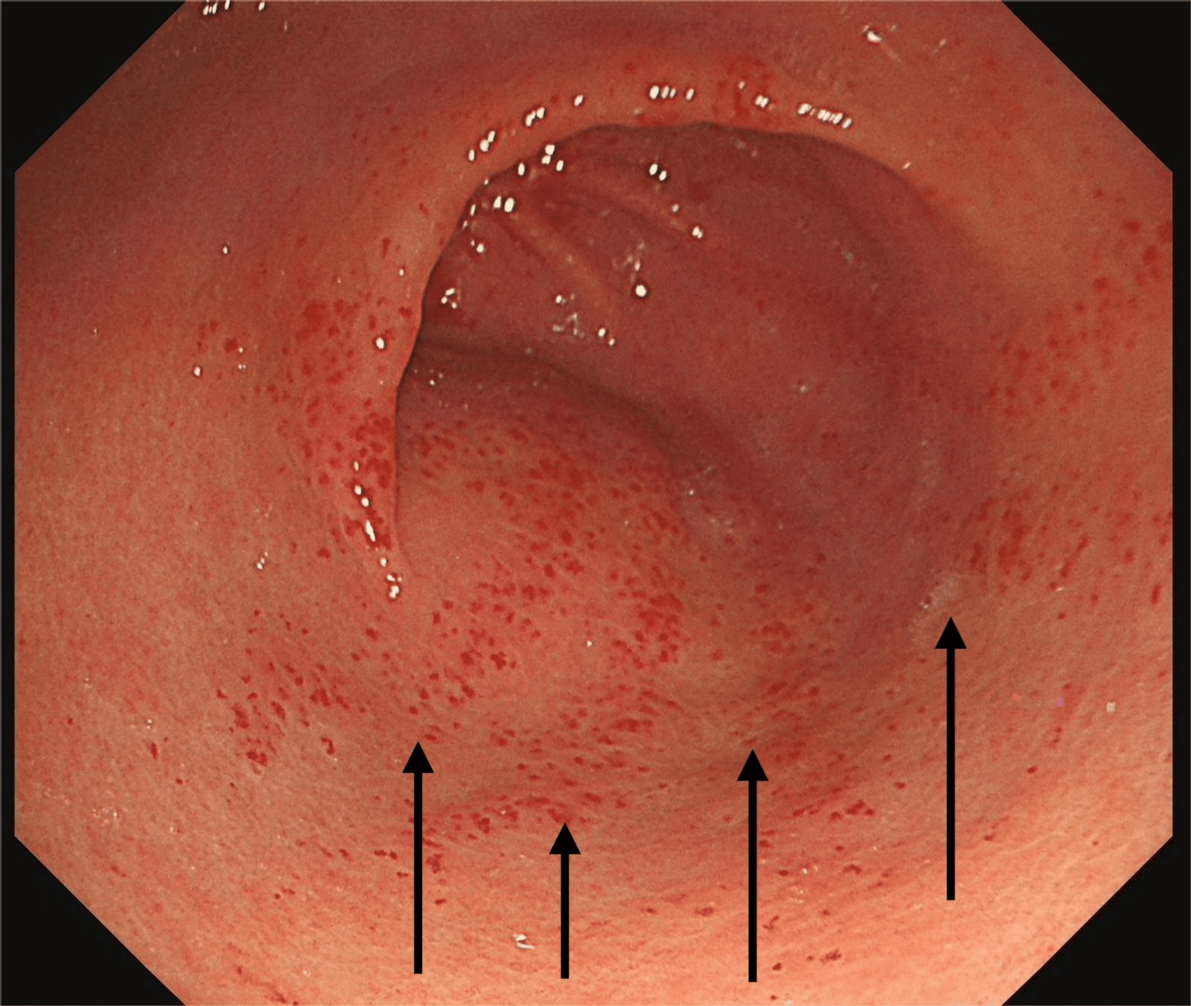
OGD (August 2025) showing GAVE with vascular ectasias in the antrum OGD: oesophago-gastro-duodenoscopy; GAVE: gastric antral vascular ectasia

**Figure 4:**
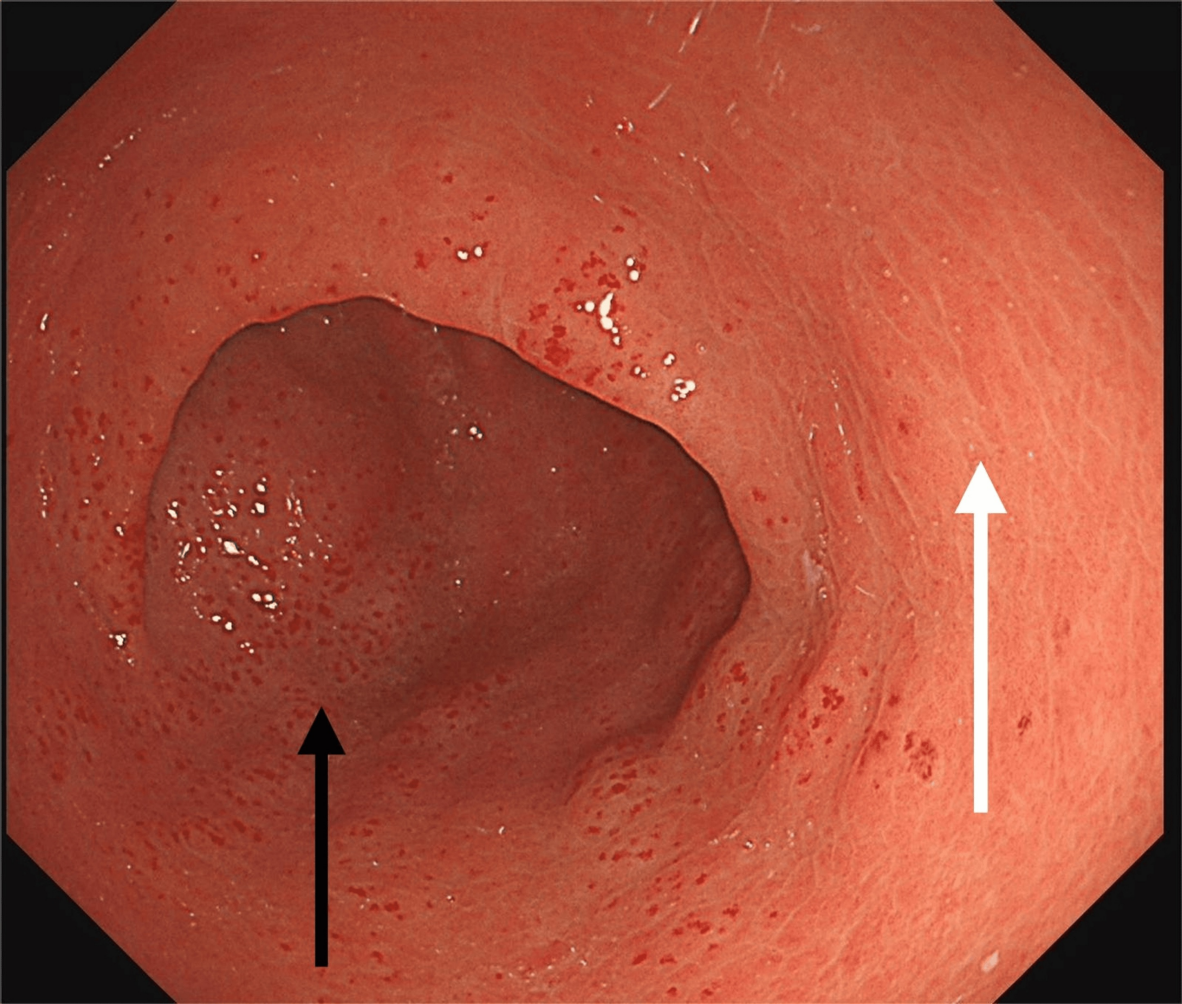
OGD (August 2025) showing combined GAVE (black arrow) and PHG with mosaic-like mucosal changes (white arrow) OGD: oesophago-gastro-duodenoscopy; GAVE: gastric antral vascular ectasia; PHG: portal hypertensive gastropathy

However, in August 2025, she re-presented with progressive shortness of breath, fatigue, and five days of dark stools. Admission blood tests showed severe microcytic anaemia with haemoglobin of 60 g/L, mean corpuscular volume (MCV) 75 fL, ferritin 7 µg/L, and transferrin saturation 6%. Repeat OGD at this admission revealed classic GAVE with active oozing (Figure 3) and areas of coexistent PHG (Figure 4).

**Figure 5:**
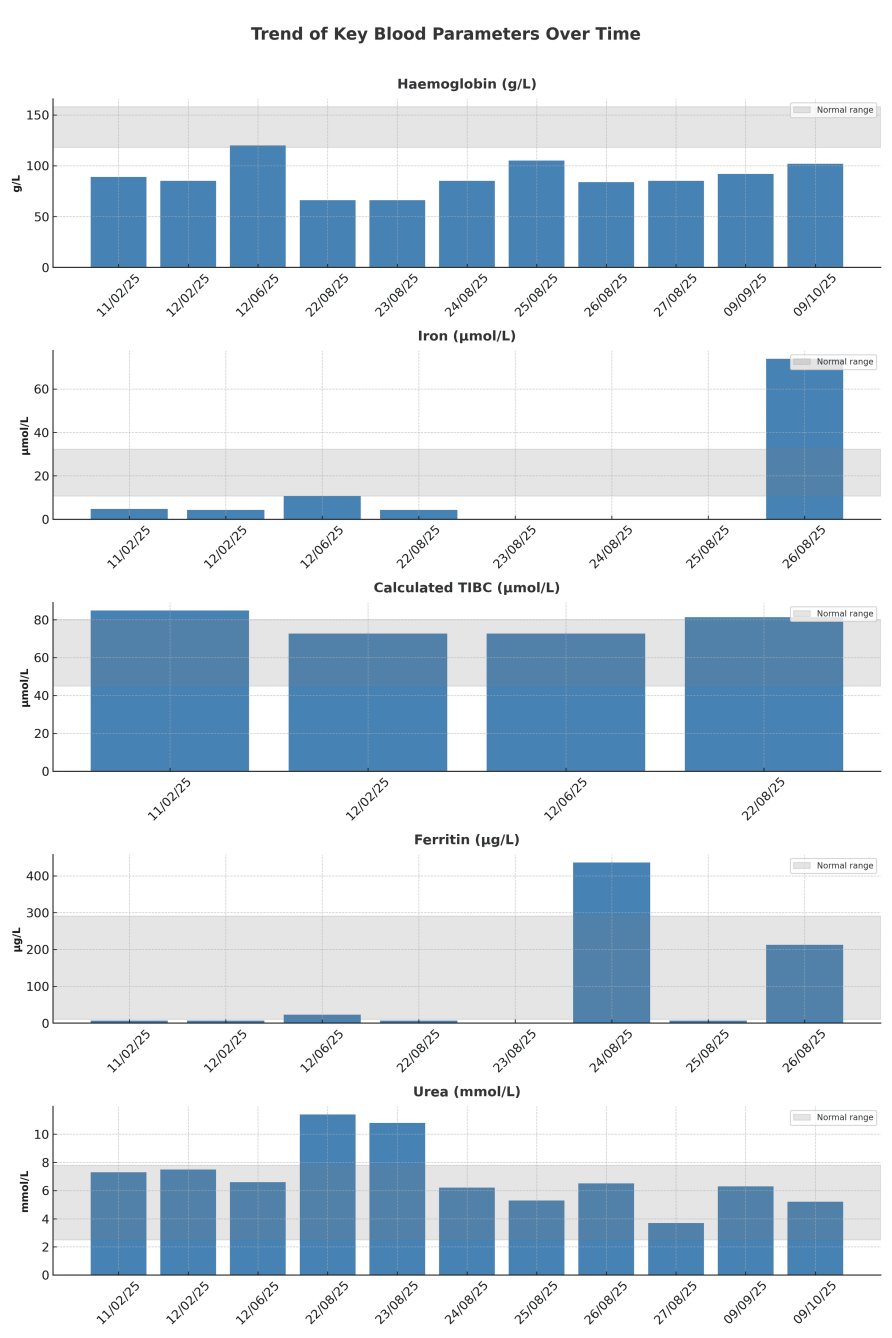
Combined graph showing the trend of haemoglobin, urea, and iron studies over time, prior and after treatment TIBC: total iron-binding capacity

Serial haemoglobin measurements during admission highlighted recurrent drops with a nadir at 60 g/L, followed by improvement after transfusion and intravenous iron infusion (Table 1 and Figure 5).

**Table 1:** Haemoglobin trend table

Request time	Material	Value (g/L)	Normal range (g/L)
27/08/2025 06:35	Blood	85 ↓	118-158
26/08/2025 09:21	Blood	84 ↓	118-158
25/08/2025 11:09	Blood	105 ↓	118-158
24/08/2025 08:26	Blood	85 ↓	118-158
23/08/2025 16:34	Blood	89 ↓	118-158
23/08/2025 00:19	Blood	66 ↓	118-158
22/08/2025 19:59	Blood	66 ↓	118-158
25/06/2025 13:02	Blood	116 ↓	118-158
12/06/2025 16:15	Blood	120	118-158
04/04/2025 14:49	Blood	119	118-158
12/02/2025 15:26	Blood	85 ↓	118-158
11/02/2025 15:07	Blood	89 ↓	118-158

Urea trends also paralleled the anaemia, reflecting fluctuating renal perfusion in the context of gastrointestinal bleeding (Table 2 and Figure 5).

**Table 2:** Urea trend table

Request time	Material	Value (mmol/L)	Normal range (mmol/L)
27/08/2025 07:01	Blood	3.7	2.5-7.8
26/08/2025 09:23	Blood	6.5	2.5-7.8
25/08/2025 11:35	Blood	5.3	2.5-7.8
24/08/2025 07:44	Blood	6.2	2.5-7.8
23/08/2025 17:06	Blood	↑ 8.2	2.5-7.8
23/08/2025 00:24	Blood	↑ 10.8	2.5-7.8
22/08/2025 19:10	Blood	↑ 11.4	2.5-7.8
14/08/2025 19:43	Blood	↑ 9.5	2.5-7.8
25/06/2025 13:25	Blood	6.7	2.5-7.8
12/06/2025 17:45	Blood	6.6	2.5-7.8
12/02/2025 17:35	Blood	7.5	2.5-7.8
11/02/2025 16:07	Blood	7.3	2.5-7.8

Iron studies confirmed profound deficiency, with iron levels as low as 4.3 µmol/L and ferritin of 7 µg/L prior to intravenous replacement. These values normalised following treatment (Tables 3-4 and Figure 5).

**Table 3:** Iron trend table

Request time	Material	Value (µmol/L)	Normal range (µmol/L)
27/08/2025 16:53	Blood	↑ 73.9	10.7-32.2
22/08/2025 19:10	Blood	↓ 4.3	10.7-32.2
12/06/2025 17:45	Blood	10.7	10.7-32.2
12/02/2025 17:35	Blood	↓ 4.8	10.7-32.2

**Table 4:** Ferritin trend table

Request time	Material	Value (µg/L)	Normal range (µg/L)
27/08/2025 17:56	Blood	213	10-291
25/08/2025 09:12	Blood	↓ 7	10-291
22/08/2025 19:10	Blood	23	10-291
12/06/2025 20:20	Blood	23	10-291
04/04/2025 16:34	Blood	↑ 436	10-291
12/02/2025 16:28	Blood	↓ 7	10-291

She received two units of red blood cells, with post-transfusion haemoglobin rising to 85 g/L, as well as intravenous ferric carboxymaltose (Ferinject). Haemostasis was achieved with PuraStat, a novel peptide gel, chosen over argon plasma coagulation (APC) due to the presence of an ICD. She was started on intravenous omeprazole in accordance with the non-variceal upper gastrointestinal bleeding protocol, as a peptic ulcer was suspected due to her ongoing aspirin therapy for cardiovascular disease; this was subsequently discontinued once a peptic ulcer was excluded. She was discharged with a structured plan for outpatient iron infusion, colonoscopy, serial blood monitoring, and hepatology follow-up.

Follow-up blood parameters demonstrated a progressive improvement in haemoglobin levels, rising from 82 g/L to 92 g/L within one month and further to 102 g/L by two months. This trend indicates a positive clinical response and successful management with PuraStat (Figure 5). Also, she underwent a follow-up colonoscopy one month later to exclude the alternative causes of IDA. The findings showed diverticulosis, with no evidence of lower gastrointestinal bleeding or other causes of IDA.

## Discussion

GAVE is a distinctive but often under-recognised cause of upper gastrointestinal bleeding and chronic IDA, particularly in elderly patients and those with comorbidities such as cirrhosis, chronic kidney disease, and autoimmune disease [1-4]. Its pathogenesis remains incompletely understood but appears multifactorial, involving mucosal trauma from gastric peristalsis, hormonal and autoimmune factors, and local mucosal hypoperfusion [3,5]. While GAVE and PHG are both associated with portal hypertension, they are now considered pathophysiologically distinct entities. GAVE is thought to result from mucosal microvascular ectasia and fibromuscular hyperplasia, whereas PHG arises from mucosal venous congestion due to elevated portal pressures [3,6]. A comparison between GAVE and PHG is given in Table 5.

**Table 5:** Comparison between GAVE and PHG GAVE: gastric antral vascular ectasia; PHG: portal hypertensive gastropathy; APC: argon plasma coagulation; RFA: radiofrequency ablation; TIPS: transjugular intrahepatic portosystemic shunt

Feature	GAVE (watermelon stomach)	PHG
Location	Gastric antrum	Gastric body and fundus
Endoscopic appearance	Longitudinal red streaks/flat red spots from the pylorus	Mosaic or snakeskin-like erythema
Histology	Dilated mucosal capillaries, fibrin thrombi, fibromuscular hyperplasia	Dilated submucosal veins from portal hypertension
Association	Cirrhosis, chronic kidney disease, and autoimmune disease; can occur without hypertension	Always linked to portal hypertension
Treatment	APC, banding, PuraStat, RFA	Portal pressure reduction (β-blockers, TIPS)

The current case demonstrates several instructive points. First, the rapid evolution of GAVE within just five months of a normal OGD emphasises that vascular gastric lesions can develop abruptly and should be re-investigated if anaemia recurs despite a recent normal endoscopy. In cirrhotic patients, mucosal vascularity and gastric blood flow can fluctuate significantly with portal pressure and systemic haemodynamics [7]. Studies have shown that the prevalence of GAVE in cirrhotics ranges between 2% and 4%, with severity correlating poorly with the degree of portal hypertension [6,8]. This dynamic nature explains the diagnostic difficulty and the potential for a normal baseline study to miss subsequent lesions.

Second, the coexistence of GAVE and PHG in this patient is unusual but clinically relevant. While each condition has been well described individually, simultaneous presentation is rare, reported in only a handful of case series [9,10]. The differentiation is crucial, as management strategies differ significantly: GAVE responds to endoscopic therapy, while PHG is best managed by reducing portal pressure (e.g., non-selective β-blockers, transjugular intrahepatic portosystemic shunt (TIPS)) [6,9]. Failure to distinguish the two may lead to suboptimal treatment and recurrent anaemia.

Third, this case highlights the diagnostic and therapeutic challenges in elderly patients with multiple comorbidities. Chronic anticoagulation, as in this case (rivaroxaban for atrial fibrillation), may exacerbate mucosal bleeding risk, while frailty and cardiac devices limit the applicability of standard treatments such as APC.

APC used to be the conventional first-line endoscopic therapy for GAVE, achieving haemostasis in approximately 80-90% of cases with significant improvement in haemoglobin levels. However, recent evidence suggests that endoscopic band ligation (EBL) may offer superior outcomes, with higher eradication rates and fewer treatment sessions, recurrent bleeding episodes, hospitalisations, and transfusion requirements compared to APC [4,5,11]. However, some lesions are too small or flat to band, and APC may be contraindicated in patients with ICDs or other high-risk settings. This creates a therapeutic gap where conventional endoscopic options are unsuitable.

PuraStat, a synthetic self-assembling peptide hydrogel, represents a promising novel alternative that acts by forming a transparent nanofibre matrix over the bleeding surface, promoting platelet aggregation and physiological healing [12,13]. Several early case series have demonstrated successful haemostasis in upper gastrointestinal bleeding, including post-endoscopic mucosal resection (post-EMR) defects, radiation proctopathy, and vascular ectasias, with good safety profiles even in anticoagulated or high-risk patients [12-14]. Its use in this case, as an alternative to APC in a patient with an ICD, underscores its emerging role in complex scenarios.

Another important aspect is the integration of iron replacement therapy into management. In GAVE-related anaemia, endoscopic therapy should always be complemented by iron repletion. Intravenous ferric carboxymaltose is preferred in elderly or intolerant patients, offering rapid correction with low risk of hypersensitivity [15]. Serial monitoring of haemoglobin, ferritin, and transferrin saturation remains essential to assess ongoing bleeding and response to therapy.

Finally, this case illustrates the need for structured follow-up and multidisciplinary coordination. Recurrent bleeding and chronic anaemia are common; long-term data suggest relapse rates up to 40-60% within two years after APC [16]. Follow-up with hepatology, gastroenterology, and haematology teams ensures the early recognition of recurrence, the optimisation of portal pressure control, and timely re-intervention. Patient education about stool colour, fatigue, and early reporting of symptoms can further reduce morbidity.

In summary, this case expands on the evolving understanding of GAVE as a dynamic mucosal vascular disorder that can coexist with PHG and present dramatically even after a normal OGD. Recognition of this overlap, use of newer haemostatic options like PuraStat, and structured long-term monitoring are key to improving outcomes in this frail, high-risk population.

## Conclusions

This case underscores the importance of maintaining a high index of suspicion for GAVE in elderly patients presenting with recurrent or severe IDA, even when recent endoscopic findings have been normal. The rapid evolution of GAVE within months highlights the dynamic vascular changes that can occur in cirrhosis and portal hypertension. The coexistence of GAVE and PHG, though rare, is clinically significant as it necessitates distinct therapeutic approaches and careful endoscopic recognition.

The successful use of PuraStat in this case demonstrates its growing utility as a novel, safe, and effective haemostatic option when traditional thermal techniques such as APC are contraindicated, for example, in patients with ICD. Optimal management requires not only targeted endoscopic therapy but also correction of iron deficiency, review of anticoagulation, and multidisciplinary coordination between gastroenterology, hepatology, and haematology teams.

Ultimately, this case highlights the need for structured long-term follow-up and proactive monitoring, as both GAVE and PHG may relapse or progress. Early recognition, tailored therapy, and vigilant surveillance can minimise morbidity, prevent recurrent bleeding, and improve overall outcomes in this vulnerable patient population.

This case illustrates the evolving therapeutic landscape of vascular gastropathies and the potential for innovative agents such as PuraStat to complement or replace conventional modalities in complex patients. Future prospective studies are warranted to compare PuraStat directly with APC and radiofrequency ablation in terms of haemostatic efficacy, recurrence rates, and cost-effectiveness. Furthermore, a greater understanding of the pathophysiological overlap between GAVE and PHG may guide more personalised management strategies, particularly in cirrhotic populations. Incorporating structured endoscopic surveillance protocols and multidisciplinary review pathways could enhance early detection and improve outcomes. Patients with cirrhosis who present with recurrent anaemia should be re-evaluated, even if the previous endoscopy was unremarkable. Emerging haemostatic agents such as PuraStat may offer an additional therapeutic option, particularly in patients who are frail, are anticoagulated, or have implanted devices, where conventional interventions may carry a higher risk. This case thereby reinforces the importance of continuous innovation, collaboration, and vigilance in the management of gastrointestinal vascular bleeding disorders.
